# Access to primary healthcare services for adults with disabilities in Latin America and the Caribbean: a review and meta-synthesis of qualitative studies

**DOI:** 10.1080/09638288.2024.2320268

**Published:** 2024-03-03

**Authors:** Veronika Reichenberger, Ana Paula Corona, Vinicius Delgado Ramos, Tom Shakespeare, Shaffa Hameed, Loveday Penn-Kekana, Hannah Kuper

**Affiliations:** a International Centre for Evidence in Disability, London School of Hygiene & Tropical Medicine, London, UK; b Department of Hearing and Speech Sciences, Multidisciplinary Institute of Rehabilitation and Health, Federal University of Bahia, Salvador, Brazil; c Instituto de Medicina Fisica e Reabilitacao, Hospital das Clinicas HCFMUSP, Faculdade de Medicina, Universidade de Sao Paulo, Sao Paulo, SP, Brazil; d Epidemiology and Public Health, Maternal and Neonatal Health Group, London School of Hygiene & Tropical Medicine, London, UK

**Keywords:** Disability, people with disabilities, primary healthcare, barriers to healthcare, Latin America and the Caribbean, meta-synthesis

## Abstract

**Purpose:**

This review and meta-synthesis of qualitative studies aims to provide an overview of qualitative evidence on primary healthcare access of people with disability in Latin America and the Caribbean, as well as to identify barriers that exist in this region.

**Methods:**

Six databases were searched for studies from 2000 to 2022. 34 qualitative studies were identified.

**Results:**

Barriers exist on both demand and supply sides. The thematic synthesis process generated three broad overarching analytical themes, which authors have related to Levesque et al.’s aspects of “ability to perceive,” “availability, accommodation and ability to reach” and “appropriateness and ability to engage.” Access to information and health literacy are compromised due to a lack of tailored health education materials. Barriers in the urban environment, including inadequate transportation, and insufficient healthcare facility accessibility create challenges for people with disabilities to reach healthcare facilities independently. Attitudinal barriers contribute to suboptimal care experiences.

**Conclusion:**

People with disabilities face several barriers in accessing healthcare. Lack of healthcare provider training, inappropriate urban infrastructure, lack of accessible transport and inaccessibility in healthcare centers are barriers that need to be addressed. With these actions, people with disabilities will be closer to having their rights met.

## Introduction

Disability cannot be regarded as simply a health issue, but if persons with disabilities do not have access to appropriate health services, they cannot enjoy their other rights. The World Health Organization estimated that 1.3 billion people lived with some form of disability in the world in 2021. In Latin America and the Caribbean (LAC), different estimates show that the regional population living with some type of disability is between 66 and 85 million, or 12–15% of the total population [1,2]. Evidence shows that on average, people with disabilities experience worse health status and outcomes than others in the population [3]. This inequity is due to exclusion and marginalization, as people with disabilities are excluded from opportunities such as education and employment [4], which can lead to worse mental and physical health [5,6]. They are also on average older and poorer and experience underlying health conditions often associated with poverty, which also contribute to worse health status. Despite their greater need for healthcare, people with disabilities face greater barriers to accessing healthcare [7–10] and consequently poorer access to healthcare [3]. Key barriers to accessing healthcare are often considered to be approachability (e.g., available information), acceptability (e.g., stigma, negative attitudes), availability (accessibility, transport), affordability (lack of insurance coverage), and appropriateness (e.g., poor skills of healthcare providers) [11].

Latin America and the Caribbean is a profoundly unequal and inequitable region, and there are crosscutting social and economic challenges that affect the access of all vulnerable populations to essential services, including healthcare [12]. While more than 218 million people are excluded from social security systems and 140 million people lack access to health services for financial, geographical or cultural reasons, people living with a disability may face additional specific barriers that are unique to their impairment [13].

Countries in the region are working to implement the progressive legal and institutional changes needed to comply with the United Nations Convention on the Rights for Persons with Disabilities [14] and regional frameworks such as the Inter-American Convention on the Elimination of all Forms of Discrimination against Persons with Disabilities [15]. To support these and other initiatives in this field, this study aims to provide an overview of the existing qualitative evidence in relation to the access of people with disability in Latin America and the Caribbean to healthcare, as well as to identify the barriers that exist in this region. Looking at barriers qualitatively will support the understanding of mechanisms and complex networks that may be missed by existing quantitative studies [16–18]. Whilst access is a multidimensional aspect of healthcare, the study adopts the framework proposed by Levesque et al. [19]. This framework uses a comprehensive approach to access by embracing both the characteristics manifested by the systems and service providers and the skills of individuals, families, and communities. The framework proposes five aspects of accessibility from both the provider and user perspectives, examining their interaction. The initial suggested dimension is “approachability” and “ability to perceive,” evaluating individuals’ capacity to recognize services, the transparency of services, health literacy, and knowledge about health. “Acceptability” and “ability to seek” consider social and cultural factors influencing access, knowledge of healthcare options, personal autonomy, and individual rights. “Availability and accommodation” and “ability to reach” encompass the physical accessibility of healthcare centers, service delivery speed, urban contexts, provider qualifications, and provider availability. On the user side, it considers transportation and mobility. “Affordability” and “ability to pay” relate to the direct costs of services and whether individuals need to pay for them. “Appropriateness” and “ability to engage” assess service quality, ensuring correct treatment and referrals, as well as patients’ ability to decide and engage autonomously [19].

## Methods

Peer-reviewed articles were retrieved from six databases (CINAHL, LILACS, MEDLINE, GLOBAL HEALTH, EMBASE CLASSIC and EMBASE) by one of the authors (first author) in June 2022. The literature search specifically sought papers published on or after the year 2000, including keywords around the concepts of access/barriers to health care services, Latin America and the Caribbean and adults with disabilities.

We use the definition of disability presented by the The United Nations (UN) Convention on the Rights for Persons with Disabilities: “Persons with disabilities include those who have long-term physical, mental, intellectual or sensory impairments which in interaction with various barriers may hinder their full and effective participation in society on an equal basis with others.” [14] Search strings used MeSH terms or equivalent headings (Appendix, supplementary material). At least two researchers consecutively screened titles, abstracts and full texts of each article initially identified for eligibility. Each of the papers were double-screened, and the third author resolved any disagreement.

Included studies were i) published in English, Spanish, Portuguese, French or Dutch (the five main official languages spoken in LAC) on or after the year 2000, ii) conducted in Latin America and the Caribbean countries, as defined by the Pan American Health Organization (PAHO), iii) investigating access to general or primary care services of adults with disabilities (including studies that investigated other age groups, but reported results for the adults separately), iv) used at least one qualitative data collection method (such as focus groups, interviews, or observations), and v) original primary research.

It excluded studies i) conducted outside of Latin America and the Caribbean, ii) investigating rehabilitation services or referral-based services (such as mental health services and other specialized care), iii) that only used quantitative data collection methods, iv) included other age groups and did not clearly differentiate the findings between them, v) commentaries, opinion pieces, letters to the editor, or conference proceedings, economic analyses, systematic reviews, project reports, policy analysis and non-peer-reviewed articles.

All selected papers were assessed for quality by two authors (first and second authors) using the consolidated criteria for reporting qualitative research (COREQ) checklist [20]. To ensure that no crucial elements are omitted, the COREQ checklist provides an extensive view of what should be incorporated into qualitative studies. This ranges from the research team, study design, data analysis and reporting [20]. No paper was excluded, however, based on quality.

The meta-synthesis was performed taking into consideration both the ENTREQ (Enhancing Transparency in Reporting the Synthesis of Qualitative Research), and the PRISMA (Preferred Reporting Items for Systematic Reviews and Meta-Analysis) [21,22]. The data extraction and coding were done by the first author of this paper and reviewed by two other researchers, including the second author of this paper. All text under the headings “results/findings” were extracted electronically and entered into *NVivo 12*. Line-by-line coding was done to find the concepts related to the Levesque framework [19].

## Results

**Figure 1:**
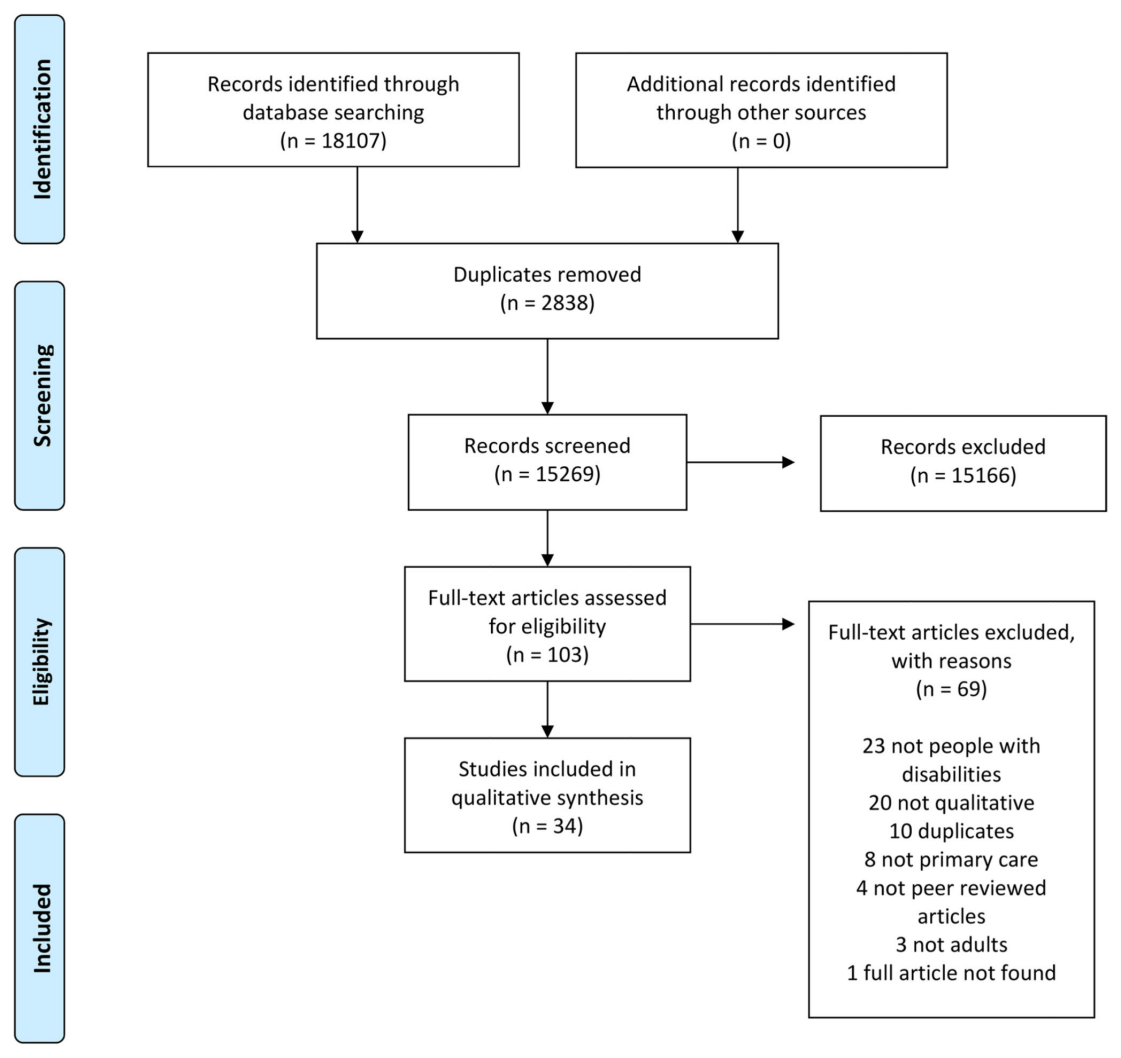
PRISMA flow chart of study identification.

The search in June 2022 identified 18,107 papers, of which 2838 duplicates were removed. 15,269 papers were double screened for titles and abstracts, with a third reviewer to resolve any disagreement. Of these, 15,166 were excluded for not meeting the eligibility criteria. One hundred and three articles full texts were reviewed, of which 69 were excluded for not meeting the eligibility criteria, including for not being peer-reviewed, being duplicates, and not being available in full. The details of the screening process involved in identifying the articles in this review are presented in Figure 1.

Of the 34 papers included, 31 were studies conducted in Brazil, two in Colombia, and one in Trinidad and Tobago. Qualitative methods used in the studies were predominantly interviews (28 studies), followed by focus groups (four studies), ethnography (one study), observation (one study) and diary (one study). Twenty-five studies included the perspective of people with disabilities, while the other nine only included non-disabled stakeholders, caregivers and/or healthcare professionals (Table 1). Ten studies focussed on access to healthcare for people with disabilities as a group, while the remaining studies focussed on specific impairment types, including 13 studies looking at people with hearing impairment, five on physical impairment, three on intellectual impairment, and three on visual impairment. The COREQ checklist analysis revealed differences in reporting quality amongst the 34 research papers. None of the studies met all the requirements on the checklist. Most of the studies reported on theoretical framework, derivation of themes, data, clarity of major themes and presented quotes (Table 2).

### Codes and analytical themes

The thematic synthesis process generated three broad overarching analytical themes, which authors have related to Levesque et al.’s aspects of “ability to perceive,” “availability, accommodation and ability to reach” and “appropriateness and ability to engage.”

#### Ability to perceive

Perceiving healthcare needs refers to access to information on health and health literacy. Most health education materials target people without visual or hearing impairments, which leaves out many people with disabilities. This review revealed that lack of accessible healthcare information and health education campaigns and opportunities affected mostly participants who have a visual impairment or a hearing impairment [37,40,43,46,50,52]. Six studies show the importance of receiving information and having accessible information on healthcare issues and how information is mostly available in text form, through posters, billboards and pamphlets, making them inaccessible to people with visual impairment [37,40,43,46,50,52]. Gaps in accessible information were found regarding sexual health, cancer prevention and oral hygiene.

For example, in one of the studies, a healthcare centre coordinator in Brazil described how the centre does not provide all the material they have in accessible forms:

We don’t have personalized material, for example, for the visually impaired, we don’t have video with [Brazilian sign language] Libras, we don’t have material in Braille and they don’t have access to information in a clear way like the general population. [43]

Lack of accessible information can enhance the vulnerability of people with disabilities [46]. Participants who could not find information from healthcare sources, such as doctors or their healthcare practice, reported seeking information from peers, primarily those with a similar impairment as their own, in the same age group, or people they know. One study reveals that people who are hearing impaired found information on sexually transmitted diseases primarily through friends and neighbours because of a lack of access to information [52]. The lack of educational resources regarding breast cancer in braille or audio recordings made it difficult for blind women to access information about their health. Women in this study revealed not feeling confident with their knowledge of self-breast exams.

Data were lacking exploring this issue for people with intellectual impairments, although they are likely to have faced informational barriers leading to difficulties in perceiving healthcare needs.

### Availability, accommodation and ability to reach

#### Urban environment

Studies show that the urban environment, in particular the surrounding areas of healthcare facilities, also contribute to barriers to accessing services [26,34–36,45,47,51,52,55,56]. Reaching primary healthcare units is not a route people with disabilities report undertaking frequently, so they must be extra careful to avoid objects, uneven pavement, or holes. A visually impaired person in Brazil noted:

At the front [of the healthcare facility] there are only steps, it’s super difficulty, and there are potholes in the street that are sometimes open and that we run the risk of falling into […] I have already fallen twice in front of the local healthcare facility. [34]

The same study shows how the main barriers for people who are visually impaired are running into obstacles in the street, as well as crossing the road alone and having difficulty taking the bus [34]. Participants who are visually or physically impaired mentioned the importance of having someone to help them to reach healthcare centres because of these difficulties with the urban environment, mostly counting on family members to accompany them [34,35,55], which creates additional barriers to accessing care.

### Transportation

Difficulties with transportation were revealed as another important barrier to accessing care. Studies show the importance of fare waivers, door-to-door transportation services, and of positive passenger and driver attitudes towards people who have a visual impairment in public transportation, such as the support of the driver who waits for him to enter the bus [56]. On the other hand, attitudes were not always so favourable, and a participant from a study conducted in Trinidad explains:

Taxi and public transportation drivers don’t treat blind persons kindly […] They just stop and would not take the extra minute to drop you in a place where it is convenient so that when you step out of the car you’re not stepping into a drain [water channel] or into the middle of the road. [36]

The distance of healthcare centres also affects participants’ ability to reach them, as the nearer the centre, the easier it is for participants to go on their own, as revealed by a study conducted in Brazil with hearing-impaired participants [35].

Some healthcare centres provide their own transportation for users, which proves to be especially important, as noted by a psychologist in a family healthcare centre in Bahia, Brazil, who explained the situation of a patient of hers who has an intellectual impairment:

Today he is no longer in receiving care because we, unfortunately, no longer have the car. The car here at the unit is broken. [26]

The lack of transportation leads to loss of follow-up and care for patients who have no other means to reach services. Additionally, adequate transportation reduces the need for the dependency of someone to accompany users to appointments and check-ups, who sometimes depend on elderly family members to take them to services [55].

### Healthcare facility accessibility

When talking about his local healthcare centre, a patient with a visual impairment in Brazil states:

The entrances are bumpy, there is no handrail, there is nothing to indicate the door, if it had those tactile floors it would be great because we would be able to orient ourselves there, and we would be safe knowing that there would be no obstacles. [34]

Other studies in Brazil revealed similarities:

There’s no ramp in the health service. They’ve already called me three times, but I can’t go, because there’s no ramp to get in; and when there is a ramp, there’s no handrail. (Physically impaired service user) [47]

My [patients] are carried by family members! Because, at the entrance door, there is no such access! There’s no reason why it’s high [the sidewalk]. Then, they usually are carried inside, we receive them in a wheelchair and arrive in my consultation room. And we do what we can do. (Nurse) [57]

People with visual impairments describe the importance of not only reaching the healthcare centre, but also getting around from room to room once they arrive at the facility. This struggle to reach rooms was also expressed by people with a physical impairment, as many facilities did not have ramps or elevators for users to reach other floors.

We have problems with Pap smears because the gynaecology room is upstairs, there is no staircase. [51]

The above quote illustrates the struggle for women with physical impairment to take necessary tests in healthcare centres that have no ramps or elevators to reach examination rooms. Studies also show the need for accessible stretchers, as the lack of accessible equipment and unsupportive staff means people are unable to undertake important health checks [34,47,51,57].

The review also revealed a lack of healthcare facility accessibility for people with a hearing impairment. A hearing-impaired man in Brazil, mentioned missing their appointments while already in the waiting room because of lack of accessible communication methods:

As a deaf person it’s difficult… I wait and wait, because I do not hear or know where to go. [43]

As illustrated above, the lack of accessible communication methods and appropriate support means, for example, that hearing-impaired patients struggle in the waiting room to know when they have been called. That creates a need for social support, which could be prevented if visual information was provided as well [29,43].

Healthcare professionals showed an awareness of the need for improved accessibility, such as accessible bathrooms, signs, elevators, ramps, and tactile flooring [26,29,30,34,45,47,51,57]. They also mentioned the importance of the availability of wheelchairs for users with mobility impairments [26] and the service being organised to meet the users’ needs [45].

### Appropriateness and ability to engage

#### Healthcare provider training

The healthcare providers expressed a general perspective that more training is needed on disability and that providing care for people with disabilities requires special assistance [25,30,40,56]. Healthcare professionals providing care for patients with spinal cord injury say, for example, they have no experience in providing care for this patient group but try to provide the best care they can [24]. Additionally, healthcare professionals described not understanding the specific care needs for people with Down Syndrome or psychosocial impairment and thinking that they will not provide the best care to them, so they prefer to refer them to other healthcare professionals [25,26].

I confess that we never received any guidance or specific training to serve this type of client, so it would be complicated and difficult to provide this type of service to a person with Down syndrome. [25]

On a similar note, a nurse in Brazil mentions her struggle in providing care to patients with a psychosocial impairment:

(…) there are a lot of patients with mental disorders and we have this difficulty calling for preventive care. So, many of them are left without doing it, because there are some that are very advanced disorders, which cannot be done! [26]

Some studies in this review reported the importance of training to improve quality of care from the perspective of users as well, who often refer to the lack of preparedness of healthcare professionals and the need for a better knowledge from professionals about people with disabilities.

A participant in a focus group in Brazil recounted a bad experience with an untrained professional:

(…) when you find some [care], it’s not good, because the professional that should be assisting people is not prepared for the [disabled] person. He opens the office door, looks at the person, gets afraid… The appointment lasts 5 minutes and he prescribes the medication without knowing the cost. [56]

This quote shows the lack of preparedness of healthcare professionals and the need for a better knowledge from professionals about people with disabilities.

#### Interpersonal quality of care

A number of studies reveal attitudinal barriers [25,40,42,51,56], including belittlement and even misdiagnosis because of prejudice, for example, not believing a physically disabled woman was pregnant:

They did a transvaginal ultrasound and saw the gestational sac, but they said it was my probe balloon. […] They went back to do a new ultrasound. The same doctor who did the other one said that he was wrong, that it was really a child, that maybe he had used his emotion instead of reason because he didn’t believe that a quadriplegic could be pregnant. [42]

Stigma was identified in the interviews with healthcare professionals in primary healthcare setting. For instance, studies revealed infantilization of people with Down Syndrome, referring to them as children, even when talking about adults [25,40]. Some participants, for example, blamed the healthcare professional’s attitude for not providing health information they sought, such as recounted by a woman with physical impairment:

When I started dating a boy aged 18–19, and my family found out, they were desperate, then they took me to the doctor and it was a horrible situation, because the gynaecologist put an anatomy book in my face, looked and said, ‘Do you see this here, you can’t use it!’ He said: ‘Look, you have everything the same, do you know what the reproductive system is? You have to know that you can’t do anything with your body.’ Those were the words he used. […] It was a very annoying situation, very painful, because I was thinking that I was going to clarify doubts because I had a lot of questions. He didn’t ask for any exam, he didn’t even think about my health. [51]

## Communication

Along with attitudinal barriers that led to health professionals not dialoguing with people with disabilities about health, this review found an additional barrier for people with a hearing impairment or a visual impairment regarding communication [29,34,35,38,43,44]. There was a fear of being deceived, fear of not understanding what is happening in the consultation because it was not explained in a way that was clear to them, including when communicating important information on therapeutic procedures and treatment plans. Participants mentioned not always feeling comfortable with having family in the consultation with them, although there was no other option. They also needed to find appointments that fit not only their schedule but the family member who was going with them, which added a layer of difficulty. Additionally, participants in the studies in this review revealed that healthcare professionals would many times direct their comments and questions to the family member instead of the individual themselves. As recounted by a hearing-impaired individual:

I wish that the deaf could go more to health services, taking care of their own health, be able to talk about what they feel about their health that could have a real communication with the professionals in health services, a real care with equality for all. [35]

One study conducted in Brazil with participants who are hearing impaired mentions healthcare professionals are not conducting their work responsibly if they are not communicating important information on therapeutic procedures and treatment plans to their hearing-impaired patients [44]. Some quotes from this paper say:

We arrive at the hospital and the doctor, nurse speaks, delivers the paper to the table, doesn’t even look properly, he has to leave the room. (P4) Sometimes I go for a gynaecological exam and I don’t know what I have. Then write in Portuguese and it’s easy for me, but I don’t understand the name of the medicine. (P6) Professionals talk fast and I don’t understand anything. (P15) When he starts talking fast I ask him to speak slowly, I’m deaf, then he says: ‘Oh, sorry.’ But then he speaks quickly again. (P16) [44]

Unfortunately, no papers were identified that explored this issue for people with intellectual impairments.

## Discussion

This is the first meta-analysis of qualitative research on access to primary healthcare for adults with disabilities in Latin America and the Caribbean. The review found evidence that people with disabilities in Latin America experience difficulties in accessing health services. We used the Levesque framework to analyse and report the findings. Our findings show that the “dimensions” and “abilities” identified as barriers were “ability to perceive,” “availability and accommodation,” “ability to reach,” “appropriateness” and “ability to engage” [19]. As the Levesque framework is not a framework used particularly to look at healthcare access for people with disabilities, some interpretations were made by the authors of this paper to fit the framework and support analysis and presentation of findings. Some findings did not fit a analytical theme of its own, such as family and social support, but is present throughout the different findings and is nuanced below. The lack of accessible information was added to “ability to perceive” as without information, people will not be able to have “health literacy” and “knowledge about health.” When looking at cultural beliefs about disability and health, Hashemi’s systematic review, which included papers from Indonesia, South Africa, among others [39], found that people with disabilities often end up seeking care from traditional healers rather than healthcare services, because of a belief that disability is related or caused by higher powers, such as religion or witchcraft. This pattern is not something that was found in this review. Religion or spiritual beliefs did not appear to get in the way of people’s healthcare-seeking attitudes in Latin America.

Consistent with previous reviews, we identified key issues with lack of accessibility of primary healthcare access [39], such as the lack of ramps. This finding is also largely supported by quantitative studies that audited healthcare facilities in the region [17,18]. Lack of accessible transportation and healthcare facility accessibility have been found as barriers in Latin America and have also been highlighted in Hashemi et al.’s review [39]. Our review shows that challenges to reaching services were caused by a lack of appropriate urban infrastructure, and the need to have someone accompanying them to healthcare services, due to the route being an uncertain one with possible obstacles. Family and social support was important when taking transportation and even going around the healthcare centre when the infrastructure was not adequate. Family and social support is a crosscutting issue identified throughout the different analytical themes and plays a key role in people’s ability to reach and engage with healthcare services. People with disabilities in our review reported the importance of a positive experience with transportation and how that supports them to go to services. Transportation is necessary because healthcare centres are not close enough to participants’ houses. A systematic review undertaken on travel time and its impact on health outcomes revealed that the relationship between worse health outcomes and travel time should be taken into account when discussing healthcare service location, as its location has a potential impact on healthcare outcomes [31].

Our review is consistent with two previous reviews in identifying a general unmet need for healthcare provider training as there was reported lack of knowledge and skills and communication difficulties [32,39]. Our review showed that some healthcare providers even expressed preferring not to provide care for people with certain disabilities because they believe they would not provide the best care for their patients. This creates an extra barrier, as primary healthcare providers are not providing the care patients need and often refer them to specialist services, hindering the continuity of care and stressing the referral pathways for those who actually need it. Patients with disabilities within our review also reported stigma from healthcare providers, misdiagnosing them or treating them disrespectfully.

Affordability was another barrier found in Hashemi et al.’s review which was not found in this review [39]. This is most likely because all countries included in our review have universal healthcare coverage, and although some affordability barriers will have arisen (such as with the indirect costs of transportation), they were not a major theme [33].

There are important programme and research implications of our findings. Service providers should enhance access to people with disabilities by providing accessible information and reasonable accommodation for people with disabilities. More training of healthcare professionals is required to support the provision of care for people with disabilities. There is also a need to improve healthcare centre accessibility, as well as local infrastructure and transportation to prevent people with disabilities from having their rights violated [14]. This would be facilitated with the strengthening of linkages between sectors, such as transportation, urban development and health. There are key research gaps remaining. There is limited evidence about healthcare access for people with intellectual impairments in Latin America and the Caribbean. Additionally, more research is required in different regions of Latin America and the Caribbean, beyond Brazil.

### Strengths and limitations of the review

A key strength of the review is that it is the first meta-analysis of qualitative research on access to primary healthcare for adults with disabilities in Latin America and the Caribbean. Moreover, we used gold-standard review methods, including use of multiple databases, dual screening, and quality assessment. Data from this study came from three groups of people: people with disabilities, their caregivers, and healthcare providers. Although most participants were people with disabilities, having information from different perspectives helped minimize gaps in the findings. Studies in multiple languages were eligible (Portuguese, Spanish, English, French, Dutch). The Levesque framework was used as a conceptual framework for the review. The review included 36 papers from a variety of literature sources, allowing us to identify themes and gain insights that would not have been possible through single studies. However, we must still be cautious and limit extrapolation, particularly since most papers are from Brazil. Certain groups were also lacking, such as people with intellectual impairments, meaning we cannot generalize the findings to all people with disabilities. The primary data from each study was inaccessible to us, so we relied on the reports from the authors included in the studies. These reported considerable variability according to their COREQ checklist but tended to be quite low.

## Conclusion

This review explored access to primary healthcare services for adults with disabilities in Latin America and The Caribbean. A number of barriers relating to both supply- and demand-side factors were identified for people with disabilities to access primary healthcare services, mostly in Brazil, but also in Colombia and Trinidad and Tobago. Limited qualitative evidence is available on healthcare access for people with disabilities in other Latin American and Caribbean countries, as well as lack of evidence on healthcare access for people with intellectual impairments. This evidence gap is important and must be filled with future research. Lack of healthcare provider training, inappropriate urban infrastructure, lack of accessible transport and inaccessibility in healthcare centres are barriers that have to be addressed for persons with disabilities to receive effective and appropriate healthcare. Persons with disabilities have a right to health under Article 25 of the Convention on the Rights of Persons with Disabilities. If these barriers can be overcome, persons with disabilities will be closer to having their rights met.

## Supplementary Material

Appendix 1

PRISMA

Supplementary file 3

## Figures and Tables

**Table 1:** Description of eligible studies.

	Study	Region, Country	Perspective	# of participants	# of participants with a disability	Type of Impairment	Data collection strategy
**1**	Tedesco et al. [53]	Porto Alegre/RS, Brazil	Healthcare professionals	13	0	Hearing	Interviews
**2**	De Miranda et al. [44]	Rio de Janeiro/RJ, Brazil	Persons with disabilities	24	24	Hearing	Focus group discussions
**3**	Oliveira et al. [35]	João Pessoa/PB, Brazil	Persons with disabilities	11	11	Hearing	Interviews
**4**	Chaveiro et al. [29]	Goiânia/GO, Brazil	Persons with disabilities	20	20	Hearing	Interviews
**5**	Fernandes et al. [57]	Vitória da Conquista/BA, Brazil	Healthcare professionals	70	0	Not specified	Focus group discussions
**6**	Castellanos Soriano et al. [58]	Bogotá, Colombia	Community leaders, elderly people of the neighbourhood, family and elderly people with disabilities	31	7	Physical	Participant observation and interviews
**7**	Ribeiro de Lago et al. [59]	Mato Grosso, Brazil	Persons with disabilities, family and healthcare professionals	5	1	Physical	Interviews
**8**	Lenardt et al. [49]	Curitiba/PR, Brazil	Caregivers	14	0	Cognitive	Interviews
**9**	de Oliveira et al. [60]	João Pessoa/PB, Brazil	Persons with disabilities	11	11	Hearing	Interviews
**10**	Gomes et al. [34]	Joao Pessoa/PB, Brazil	Persons with disabilities	24	24	Visual	Interviews
**11**	Ianni et al. [30]	São Paulo/SP, Brazil	Persons with disabilities, caregivers, stakeholders	25	2	Hearing	Interviews
**12**	Santos et al. [42]	Rio de Janeiro/RJ, Brazil	Persons with disabilities	6	6	Physical	Interviews
**13**	Fontanella et al. [40]	São Carlos/SP, Brazil	Healthcare professionals	16	0	Cognitive	Interviews
**14**	Pereira et al. [50]	Natal/RN, Brazil	Persons with disabilities	30	30	Hearing	Interviews
**15**	Cruz et al. [38]	Campina Grande/ PB, Brazil	Persons with disabilities	16	16	Visual	Questionnaires with open questions
**16**	Mattevi et al. [56]	Porto Alegre/RS, Brazil	Persons with disabilities and caregivers	23	15	Physical, cognitive, visual, multiple	Focus group discussions
**17**	Fiorati et al. [23]	Ribeirão Preto/SP, Brazil	Persons with disabilities	10	10	Physical, cognitive, multiple	Interviews
**18**	Nicolau et al. [51]	São Paulo/SP, Brazil	Persons with disabilities	15	15	Physical, visual, hearing, cognitive	Interviews
**19**	Costa et al. [46]	Minas Gerais/MG, Brazil	Persons with disabilities	9	9	Hearing	Interviews
**20**	Bentes et al. [52]	Crato/CE, Brazil	Persons with disabilities	12	12	Hearing	Interviews
**21**	Santos Sales et al. [26]	Bahia/BA, Brazil	Healthcare professionals, social workers	9	0	Not specified	Interviews
**22**	Aokia et al. [55]	São Paulo/SP, Brazil	Persons with disabilities	5	5	Physical, cognitive and multiple	Interviews
**23**	Franca et al. [24]	Paraiba, Brazil	Healthcare professionals	20	0	Spinal cord injury	Interviews
**24**	Carvalho et al. [28]	Natal/RN, Brazil	Persons with disabilities	12	12	Physical	Interviews
**25**	Miranda et al. [25]	Rio Grande do Norte, Brazil	Healthcare professionals	12	0	Cognitive	Interviews
**26**	Othero et al. [45]	São Paulo/SP, Brazil	Healthcare professionals	6	0	Not specified	Interviews
**27**	Dubow et al. [54]	Rio Grande do Sul, Brazil	Healthcare professionals and stakeholders	49	0	Not specified	Interviews
**28**	Monteiro et al. [48]	Rio Grande do Norte, Brazil	Persons with disabilities	30	30	Visual	Interviews
**29**	Gil Cano et al. [37]	Medellin, Colombia	Persons with disabilities	12	12	Hearing	Focus group discussions and questionnaires
**30**	Fernandes et al. [41]	Fortaleza/CE, Brazil	Persons with disabilities and teachers	40	20	Hearing	Participant observations and questionnaire
**31**	Castro et al. [47]	São Paulo/SP, Brazil	Persons with disabilities	25	25	Physical, visual, hearing	Semi-structured questionnaire
**32**	Santos et al. [27]	Rio de Janeiro, Brazil	Persons with disabilities	121	121	Hearing	Mixed-methods; interviews
**33**	Cardoso et al. [43]	Goiânia/GO, Brazil	Persons with disabilities	11	11	Hearing	Interviews
**34**	Parey et al. [36]	Trinidad, Trinidad and Tobago	Persons with disabilities	26	26	Physical, cognitive, sensory, multiple	Interviews

**Table 2:** Comprehensiveness of reporting using the COREQ checklist.

No	Item	No of studies (*n* = 34)	Research studies
**Domain 1: Research team and reflexivity**			
Personal Characteristics			
1	Interviewer/facilitator	7	[6–12]
2	Credentials	15	[7–9,12–23]
3	Occupation	10	[6–9,11,12,14,15,24,25]
4	Gender	23	[6–14,16,17,19,20–29,34]
5	Experience and training	8	[8,9,10,13,15,21,27,34]
Relationship with Participants			
6	Relationship established	1	[9]
7	Participant knowledge of the interviewer	1	[9]
8	Interviewer characteristics	7	[8,9,10,15,21,27,34]
**Domain 2: study design**			
Theoretical framework			
9	Methodological orientation and Theory	31	[1–3,6–32,34]
Participant selection			
10	Sampling	28	[1–12,14,16–18,20–22,24–29,31,32,34]
11	Method of approach	14	1,2,3,4,7,8,10,14,17,23,26,27,29,34
12	Sample size	34	[1–34]
13	Non-participation	4	[4,5,27,28]
Setting			
14	Setting of data collection	23	[1–11,13–18,22,23,25–29,34]
15	Presence of non-participants	8	[4,9,11,15,17,18,23,27]
16	Description of sample	26	[2–5,7–17,20–23,25–29,31,34]
Data Collection			
17	Interview guide	24	[1,5,6,9,10,11,13–19,21–25,28–32,34]
18	Repeat interviews	0	
19	Audio/visual recording	21	[2,5,9,10,12,14,15,17–19,21,23,25,26,28–34]
20	Field notes	2	[7,27]
21	Duration	6	[6,10,19,26,27,34]
22	Data saturation	12	[1,2,3,5,12,15,17,21,28,29,31,34]
23	Transcripts returned	3	[7,14,34]
**Domain 3: Analysis and findings**			
Data analysis			
24	Number of data coders	3	[10,31,34]
25	Description of the coding tree	29	[4–13,14,15,17–34]
26	Derivation of themes	31	[1,2,5–33]
27	Software	4	[3,11,29,25]
28	Participant checking	2	[30,34]
Reporting			
29	Quotations presented	31	[2–30,32,34]
30	Data and findings consistent	33	[1–30,32–34]
31	Clarity of major themes	31	[2–30,32–34]
32	Clarity of minor themes	1	[2]

## ➤ Implications for Rehabilitation

- The identification of barriers on both the supply and demand sides highlights implications for individuals with disabilities seeking access to primary healthcare services, primarily in Brazil, with similar concerns noted in Colombia and Trinidad and Tobago.
- Service providers should enhance access to people with disabilities by providing accessible information and reasonable accommodation for people with disabilities.
- More training of healthcare professionals is required to support the provision of care for people with disabilities.
- There is a need to improve healthcare centre accessibility, as well as local infrastructure and transportation to prevent people with disabilities from having their rights violated.
- Linkages should be strengthened between sectors like transportation, urban development, and health to enhance overall accessibility and prevent violations of the rights of individuals with disabilities.
