# Supplementary material for: Access to primary healthcare services for adults with disabilities in Latin America and the Caribbean: a review and meta-synthesis of qualitative studies
Source: Disabil Rehabil. Author manuscript; Available in PMC 2024 Sep 19. (PMC7616503; doi:10.1080/09638288.2024.2320268)
Supplement: Appendix 1 [file EMS194604-supplement-Appendix_1.docx]

Appendix 1 Search terms

| **#** | **Searches** | **Annotations** |
| --- | --- | --- |
| 1 | (Caribbean or Latin America or Central America or South America).ti,ab. |  |
| 2 | exp caribbean region/ or exp central america/ or latin america/ or exp south america/ |  |
| 3 | (Guadaloupe or Aruba or Martinique or Turks or Caicos or Turks and caicos islands or virgin islands or Peru or Argentina or Brazil or Chile or Colombia or Venezuela or Cayman Or Puerto Rico Or Saint Barthelemy or ST Barthelemy or Guatemala or Ecuador or Bolivia or Haiti or Cuba or Dominican Republic or Honduras or Paraguay or Nicaragua or El Salvador or Costa Rica or Panama or Uruguay or Jamaica or Trinidad or Tobago or Guyana or Suriname or Belize or Bahamas or Barbados or St Lucia or Saint Lucia or Grenada or St Vincent or Saint Vincent or Grenadines or Antigua and Barbuda or Dominica or Saint Kitts or St Kitts or Nevis).mp. |  |
| 4 | 1 or 2 or 3 | Latin America and the Caribbean |
| 5 | (disable* or Disabilit* or Handicap*) adj5 (person* or people))).sh,ti,ab. |  |
| 6 | (Physical* adj5 (impair* or deficienc* or disable* or disabili* or handicap*)).mp. |  |
| 7 | (Cerebral pals* or Spina bifida or Muscular dystroph* or Arthriti* or Osteogenesis imperfecta or Musculoskeletal abnormalit* or Musculo-skeletal abnormalit* or Muscular abnormalit* or Skeletal abnormalit* or Limb abnormalit* or Amputation* or Clubfoot or Poliomyeliti* or Paraplegi* or Paralys* or Paralyz* or Hemiplegi*).mp. |  |
| 8 | ((Hearing or Acoustic or Ear$3) adj5 (loss* or impair* or deficienc* or disable* or disabili* or handicap*)).mp. |  |
| 9 | ((Visual* or Vision or Eye$3) adj5 (loss* or impair* or deficienc* or disable* or disabili* or handicap*)).mp. |  |
| 10 | (Deaf* or Blind*).mp. |  |
| 11 | exp Hearing impairment/ or exp vision disorders/ or exp Deafness/ or exp Blindness/ |  |
| 12 | (Schizophreni* or Psychosis or Psychoses or Psychotic Disorder* or Schizoaffective Disorder* or Schizophreniform Disorder* or Dementia* or Alzheimer*).mp. |  |
| 13 | exp "schizophrenia and disorders with psychotic features"/ or exp Dementia/ or exp Alzheimer disease/ |  |
| 14 | ((Intellectual* or Mental* or Psychological* or Developmental) adj5 (impair* or retard* or deficienc* or disable* or disabili* or handicap* or ill?6)).mp. |  |
| 15 | ((communication or language or speech or learning) adj5 disorder* or disabilit*).mp. |  |
| 16 | (Autis* or Dyslexi* or Down* Syndrome or Mongolism or Trisomy 21).mp. |  |
| 17 | exp Learning Disorders/ |  |
| 18 | exp Mentally Disabled Persons/ |  |
| 19 | 17 or 18 |  |
| 20 | Or/5-18 | Disability search terms |
| 21 | exp Immunization/ |  |
| 22 | exp Immunization Programs/ |  |
| 23 | exp Health Services/ |  |
| 24 | exp Mass Screening/ |  |
| 25 | exp health program/ |  |
| 26 | exp health care/ |  |
| 27 | access*.mp. |  |
| 28 | health*.mp. |  |
| 29 | equal*.mp. |  |
| 30 | inequal*.mp. |  |
| 31 | barrier*.mp. |  |
| 32 | exp Preventive Health Services/ or exp Health Services for Persons with Disabilities/ or exp Primary Health Care/ or exp Health Promotion/ |  |
| 33 | exp Palliative Care/ |  |
| 34 | palliat*.mp. |  |
| 35 | promot*.mp. |  |
| 36 | 33 or 34 | Palliative care |
| 37 | health promot*.mp. |  |
| 38 | 35 or 37 | Health promotion |
| 39 | prevent*.mp. |  |
| 40 | 21 or 22 or 24 or 39 | Screening, immunization, prevention |
| 41 | 23 or 25 or 26 or 28 | ‘Health’ |
| 42 | 36 or 38 or 40 or 41 | Health care (prevention, promotion, palliative) |
| 43 | (access* or afford* or accept* or avail* or "geographic avail*").mp. | Access |
| 44 | facilitat*.mp. |  |
| 45 | 31 or 44 | Barrier or facilitator |
| 46 | 29 or 30 or 43 or 45 | Access, barrier/facilitator, inequalities |
| 47 | 42 and 46 |  |
| 48 | 4 and 20 and 47 | Final search |
| 49 | Limit 48 to yr="2000 -Current" | Final search between 2000-Current |
